# The magnitude of casual sex and associated factors among students at Debre Berhan University

**DOI:** 10.3389/frph.2024.1491617

**Published:** 2024-12-19

**Authors:** Tsega Mathewos, Esubalew Tesfahun, Muluken Tessema Aemiro, Tadesse Mamo Dejene

**Affiliations:** ^1^ Department of Public Health, School of Public Health, Asrat Woldeyes Health Science Campus, Debre Berhan University, Debre Birhan, Ethiopia; ^2^ Department of Epidemiology, Biostatistics and Health Informatics, School of Public Health, Asrat Woldeyes Health Science Campus, Debre Berhan University, Debre Birhan, Ethiopia; ^3^ Department of Public Health, School of Public Health, Asrat Woldeyes Health Science Campus, Debre Berhan University, Debre Birhan, Ethiopia

**Keywords:** casual sex, students, Debre Berhan University, associated factors, Ethiopia

## Abstract

**Background:**

Today's youth are adopting a new trend: casual sex. College students are expected to engage in a range of potentially harmful sexual activities, such as casual sex. Numerous factors can lead students to engage in casual sexual encounters. However, there has been limited research on the prevalence of casual sex and related issues in higher education institutions in Ethiopia.

**Objective:**

To assess the prevalence of casual sex and its associated factors among regular undergraduate students at Debre Berhan University in 2023.

**Methods:**

From 15 May 2023, to 30 May 2023, regular undergraduate students from Debre Berhan University participated in a cross-sectional study. A total of 512 samples were collected using a multistage sampling technique. Subsequently, a simple random sampling technique was employed to select the students, with a proportionate allocation of samples distributed to randomly selected colleges, departments, and academic years. The data were collected using a structured, pretested, and self-administered questionnaire. Once the data were imported into Epi-data version 4.6 and exported to SPSS version 20 for analysis, frequencies, percentages, and tables were generated. Bivariate and multivariate logistic regression models were used to identify characteristics associated with casual sex.

**Results:**

The prevalence of casual sex among regular undergraduate students at Debre Berhan University was 31.0% 95% CI (27.2–34.6). Several factors were significantly associated with casual sex. These included students from urban areas [AOR: 2.95; 95% CI (1.66–5.24)], students with poor academic performance [AOR: 2.81; 95% CI (1.51–5.22)], alcohol consumption [AOR: 4.59; 95% CI (2.60–8.12)], attending nightclubs [AOR: 2.75; 95% CI (1.47–5.16)], watching pornography [AOR: 2.59; 95% CI (1.47–5.16)], and peer pressure [AOR: 2.24; 95% CI (1.38–3.65)].

**Conclusion:**

The prevalence of casual sex was high among Debre Berhan University students. In order to lessen the negative effects of casual sex practices, this study found predictors that can be avoided through various interventions. Key preventative measures include improving student academic performance, providing engaging and free entertainment, restricting access to pornographic websites on university Wi-Fi, and employing peer educators.

## Introduction

The term “casual sex” refers to sexual activity that takes place outside of a romantic relationship or marriage and is typically engaged in without any expectations of commitment or exclusivity (1).

Casual sexual relationships or encounters are defined or referred to in various ways in different research literature. These include “sex with no strings attached,” which encompasses situations such as a one-night stand or a hookup. This typically occurs when two individuals, who may be strangers or friends, meet at a party, social gathering, or bar and end up having a single sexual encounter (2). Friends with benefits (FWB) or “fuck buddies” are terms used to describe two friends who engage in sexual activity, while both understand that they are not in a committed or monogamous relationship (3). A “booty call” is a more recent concept where individuals can call or text to see if the other person is available or interested in meeting up for sex. “Sex with an ex-boyfriend or ex-girlfriend” is another form of casual sex. If individuals are extremely aroused or feel particularly lonely, they might call their ex for sex (4).

Casual sex is categorized as a risky sexual behavior. The risks associated with casual sex are often linked to a lack of knowledge about the partner's sexual history or an inability to communicate about this and sexual safety, often due to embarrassment or intoxication (2). Casual sex might occur between partners just once or on a regular basis, and it could be scheduled in advance or happen spontaneously (5).

Since university students fall into the youth age category, they are more susceptible to engaging in sexual activities outside of a romantic relationship. This can lead to risks such as HIV/AIDS, sexually transmitted diseases, depression, unintended pregnancy, and abortion (6).

There is limited evidence on casual sexual relationships in Ethiopia. Therefore, this study aimed to assess the prevalence and factors associated with casual sex among undergraduate students at Debre Berhan University.

## Methods and materials

An institution-based cross-sectional study was conducted from 15 May to 30 May 2023, at Debre Berhan University. The main campus is located 1 kilometer away from the center of Debre Berhan town, while the Asrat Woldeyes Health Science Campus is closer to the city. According to the 2023 registrar's office report, DBU has approximately 19,383 students. Of these, 6,759 were regular students, 2,983 were extension students, 8,462 were summer students, and 1,179 were enrolled in continuing and distance education programs. Currently, the university comprises 8 colleges.

### Inclusion and exclusion criteria

The study included regular undergraduate students from Debre Berhan University who were attending the university at the time of data collection in those four colleges. However, students who were unable to respond due to illness during the data collection period were excluded.

### Sample size determination

The sample size for the current study is estimated for each specific objective by using a single proportion formula.

✓ **Specific objective one**: Magnitude of casual sex

By assuming a 28% prevalence of casual sex from a study performed in Dessie town (7) with a 95% confidence interval and 5% desired precision and adding a 10% nonresponse rate and design effect of 1.5

$$n=\frac{{\left(Z_{\alpha /2}\right)}^{2}\times \;P(1-P)}{d^{2}}\;n=\frac{{\left(1.96\right)}^{2}(0.28\times 0.72)}{(0.05{)}^{2}}=309.78=310$$

✓ **Specific objective two**: Factors associated with casual sex were calculated (Table 1)

**Table 1:** Sample size determination for the factors associated with casual sex.

Outcome variable	Associated factors	CI	Power	AOR	Total sample size
Magnitude of casual sex	Peer pressure	95%	80%	8	258
	Alcohol drinking	95%	80%	7.7	220
	Attending night club	95%	80%	4	198
	Watching pornography	95%	80%	2.8	195

The sample sizes for the two objectives were compared, and the largest sample size was 310; considering a nonresponse rate of 10% and a design effect of 1.5, the final sample size was 512.

### Sampling technique and procedure

**Figure 1:**
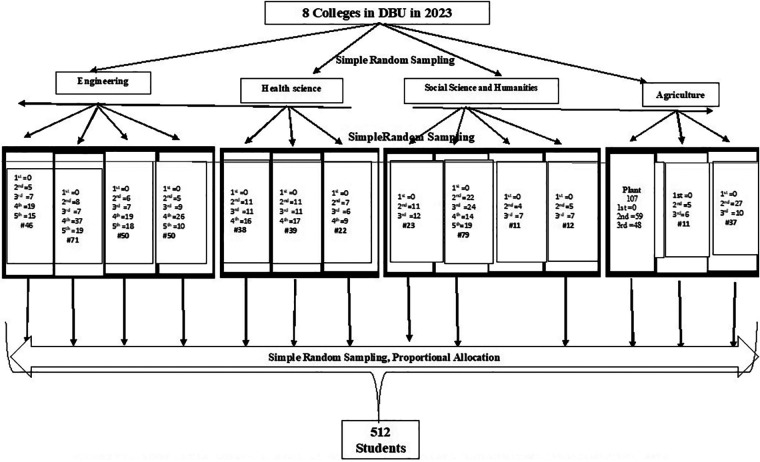
Schematic presentation of the sampling procedure for regular undergradu are students in Debre Berhan University, 2023.

A multistage selection process was used to choose a student sample that was approximately representative of the eight universities that comprise DBU University. Four colleges were chosen by lottery in the first round. Following the acquisition of a list of departments, 14 departments from these universities were chosen for the second phase using a straightforward random sample technique. The pupils were then placed in groups according to their academic year. Ultimately, study participants were chosen for each year of the program using a basic random sampling technique, moving from dorm to dorm and courses to dorm (Figure 1).

### Data collection method and procedures

A structured questionnaire was adapted from the CDC's Youth Risk Behavior Surveillance System (YRBSS) (8). Data collectors received training that included understanding the study's general goal and becoming acquainted with the instrument used to gather participant responses. 25 students who were not research participants in another region were pretested in order to ensure the data collecting tool's consistency and clarity. The tool's consistency and dependability were then assessed employing the Cronbach's alpha test (0.75), which shows that the instrument is adequate for evaluating the study's necessary variables.

The relevant literature was also reviewed and organized according to the specific objectives it could address. The questionnaire consists of three parts: information on the sociodemographic of selected students, information on the prevalence of casual sex, and information on factors associated with casual sex. The data were collected through self-administered techniques using a well-structured questionnaire. The data collection team consisted of two clinical nurses and one supervisor with a BSc in public health.

### Operational definitions

#### Casual sex

Regular undergraduate students with DBU experience at least one of the following: having multiple sexual partners, sex with commercial sex workers, sex for incentives, sex between best friends/FWB, sex with ex-boy/girlfriend in the last 12 months (1–5).

#### Ever had sex

Individuals who experience penile-vaginal sexual intercourse in their lifetime.

#### Multiple sexual partners

Having more than one sexual partner in the last 12 months.

#### Substance use

The use of at least one of the following substances, alcohol, Khat, or cigarettes, which are assumed to affect one's level of thinking and increase one's chance of engaging in casual sex in the last 12 months.

#### Sex for incentive

Having sex with someone to earn money or other benefits in the last 12 months.

#### Peer pressure

In this study, it was referred to as a subjective feeling of being pushed, urged, or dared by others to engage in sexual activity with a casual person in the last 12 months.

### Data processing and analysis

The investigator entered the data using Epi-data version 4.6 statistical software and exported it to SPSS version 20 for analysis when the data collection was finished. The data were then reviewed for consistency and completeness. The results were summarized using descriptive analysis (frequency, mean, and proportion). Tables displaying the results are displayed. To ascertain the relationship between predictors and outcome variables and to manage cofounders, bivariate and multivariate logistic regression models were used. In the bivariate logistic regression model, variables with a *P* value less than 0.25 were included. Associations with a *p* value <0.05 were deemed to be statistically significant after a multivariable logistic regression model was used.

### Data quality management

Initially, the questionnaire was prepared in English, translated into Amharic, and then translated back to English to evaluate its consistency. Before the actual data collection, the questionnaire was pretested on 25 students at a college who were not included in the sample. Necessary amendments, such as ordering, skipping patterns, and ensuring the time required to complete the questions, were made based on the feedback obtained from the pretest. The data collectors and supervisor were trained for one day on the objectives and methodology of the research, as well as issues associated with the self-administered questionnaire, including the need for proper categorization and coding of the response variables. This training was conducted by the principal investigator. Because it impacts the individual variable contribution of the overall interest, multicollinearity is an issue in regression. For this reason, multicollinearity was tested using the variance inflation factor (VIF), and the result was 4.362, indicating that there is no multicollinearity effect among the independent predictors of casual sex, its model fitness was assessed using the Hosmer-Lemeshow test (*P* value = 0.071), which shows that the model fits the data because the *p* value is negligible, an indication of the model's strong fitness.

## Results

### Socio demographic characteristics

A total of 500 regular undergraduate students participated in this study, for a response rate of 97.6%. More than half of the respondents, 258 (51.6%), were males. The overall mean age of the study participants was 22.36 (±1.96) years. The majority of the participants, 80.8%, were enrolled at the Debre Berhan University main campus. Additionally, 69.6% of the participants came from an urban area, 61.6% had pocket money ranging from 0 to 1000 birrs, and 45.8% attended churches or mosques once per week (Table 2).

**Table 2:** Sociodemographic characteristics of regular undergraduate students at Debre Berhan University, 2023.

Variables	Category	Frequency	Percent (%)
Sex	Male	258	51.6
	Female	242	48.4
Age in Years	≤22	271	54.2
	>22	229	45.8
Marital status	Single	473	94.6
	Married	27	5.4
College	Nonhealth	404	80.8
	Heath	96	19.2
Year of study	2nd year	155	31
	3rd year	149	29.8
	4th year	116	23.2
	5th year	80	16
Cumulative GPA	≤2.5	81	16.2
	>2.5	419	83.8
Frequency attend on religion places	Every day	146	29.2
	Once per week	229	45.8
	Once per month	72	14.4
	Once per year	53	10.6
Residence	Rural	152	30.4
	Urban	348	69.6
Accommodation	Dormitory	419	83.8
	With parents/relatives	58	11.6
	Rented	23	4.6
Pocket money	0–1,000	308	61.6
	1,001–2,000	135	27
	2,001–3,000	36	7.2
	>3,001	21	4.2

### Sexual experiences of the study subjects

Of the total study participants, three-fourths, or 373 (74.6%), reported having a history of previous sexual intercourse. Among those, 223 (59.8%) had their first sexual intercourse before the age of 18. The majority, 229 (61.4%), had their first sexual intercourse before joining the university, and 259 (69.4%) had their first sexual intercourse with their boyfriends or girlfriends. The reason for the first sexual encounter was falling in love for 160 (42.9%) of the students. Among the total participants who had a history of sexual intercourse, 174 (46.6%) of the respondents had never used a condom during sex. Among those participants who did not use condoms, the major reason given was that condoms decrease sexual satisfaction, according to 105 (37.9%) of the respondents (Table 3).

**Table 3:** Sexual experience of regular undergraduate students of Debre Berhan University, 2023.

Variables	Category	Frequency	Percent (%)
Ever had a sexual experience	Yes	373	74.6
	No	127	25.4
Age at first sex	≤18	223	59.8
	>18	150	40.2
When do you have your first sex?	Before joining university	229	61.4
	After joining university	144	38.6
With whom you have your first sex	Steady boy girlfriend	259	69.4
	Casual boy girlfriend	72	19.3
	Husband/wife	20	5.4
	Commercial sex worker	8	2.1
	Other (best friend)	14	3.8
Reason for making your first sexual intercourse	Had desire	60	16.1
	Fell in love	160	42.9
	Got married	19	5.1
	Peer pressure	58	5.5
	Got drunk	69	18.5
	To get money or another gift	7	1.9
Condom utilization	Yes	199	53.4
	No	174	46.6
Frequency of condom utilization	Always	96	25.8
	Some times	103	27.6
	Never	174	46.6
Reason for not using a condom	Difficult to get a condom	16	5.8
	Too expensive	10	3.6
	Decreases sexual satisfaction	105	37.9
	Trust partner	63	22.7
	Embarrass to buy	32	11.6
	Partner rejection	27	9.8
	I got drunk	24	8.7

### Magnitude of casual sex

**Figure 2:**
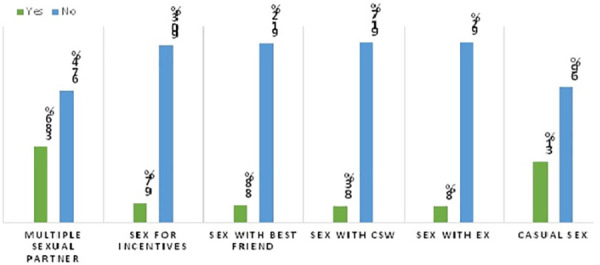
The magnitude of casual sex among regular undergraduate students of Debre Berhan University students in 2023.

Among the total participants who had a history of sexual intercourse, 38.6% of the respondents reported having more than one sexual partner in the last 12 months. Additionally, 9.7% of respondents practiced sex to receive incentives. Furthermore, 8.8%, 8.3%, and 8% of the respondents had sex with their best friend, a commercial sex worker, and their previous or ex-boyfriend/girlfriend, respectively. The overall prevalence of casual sex among regular undergraduate students at Debre Berhan University was 31.0%, with a 95% confidence interval (CI) of 27.2–34.6% (Figure 2).

### Predisposing factors among the study subjects

Among all the students, 200 (or 40%) were subjected to peer pressure, 292 (58.4%) drank alcohol, 82 (16.4%) chewed khat, 43 (8.6%) smoked, 186 (37.2%) watched pornography, 337 (67.4%) had previously attended a nightclub, and 470 (94%) used social media (Table 4).

**Table 4:** Predisposing factors among regular undergraduate students at Debre Berhan University, 2023.

Predisposing factors	Frequency	%
Peer pressure	200	40
Alcohol drinking	292	58.4
Chewing khat	82	16.4
Cigarette smoking	43	8.6
Watching pornography	186	37.2
Night club	337	67.4
Social media	470	94

### Effect of associated factors on the magnitude of casual sex in the study subjects

The following were candidate factors in the bivariable logistic regression analysis: age, CGPA, residency, pocket money, peer pressure, alcohol use, khat chewing, pornography, nightclubs, and social media use. Multivariate logistic regression analysis revealed that casual sex was strongly correlated with peer pressure, CGPA, residence, alcohol consumption, pornography viewing, and club attendance. Students with a cumulative GPA below 2.5 were 2.81 times more likely than those with a CGPA over 2.5 to be involved in casual sexual activity [AOR: 2.81; 95% CI (1.51–5.22)].

Compared to students from rural areas, those from urban areas had a 2.95-fold greater likelihood of participating in casual sex [AOR: 2.95; 95% CI (1.66–5.24)]. Compared to research participants who did not experience peer pressure, students who experienced peer pressure to have sex were 2.24 times more likely to engage in casual sex [AOR: 2.24; 95% CI (1.38–3.65)]. According to this study, students who consumed drugs had a 4.59-fold increased risk of engaging in casual sex [AOR: 4.59; 95% CI (2.60–8.12)]. Compared to students who never went to nightclubs, students who frequented them had a 2.75 times greater likelihood of engaging in casual sex [AOR: 2.75; 95% CI 2.48 (1.47–5.16)].

The study subjects who watched pornography were 2.59 times more likely to engage in casual sex than those who did not have such a history. [Adjusted odds ratio (AOR): 2.59; 95% confidence interval (CI): 1.47–5.16] (Table 5).

**Table 5:** Multivariate analysis of factors associated with the magnitude of casual sex among regular undergraduate students at Debre Berhan University, 2023.

Variables	Category	Casual sex		COR 95% CI	AOR 95% CI	*P* value
		**Yes**	**No**			
Age in Years	≤22	66	205	1	1	1
	>22	89	140	1.98 (1.35–2.90)	1.20 (0.67–2.19)	0.540
CGPA	≤2.5	41	39	2.92 (1.79–4.74)	**2.81** **(1.51–5.22)**	0.001*
	>2.5	114	306	1	1	1
Residence	Rural	22	130	1	1	1
	Urban	133	215	3.66 (2.22–6.03)	**2.95** **(1.66–5.24)**	**0.000**
Pocket Money	0–1,000	89	219	1	1	1
	1,001–2,000	39	96	1 (0.64–1.56)	0.59 (0.34–1.04)	0.068
	2,001–3,000	15	21	1.76 (0.88–3.55)	1.01 (0.42–2.40)	0.988
	>3,001	12	9	3.28 (1.37–8.06)	2.36 (0.62–8.94)	0.205
Peer pressure	Yes	81	119	2.08 (1.41–3.06)	**2.24** **(1.38–3.65)**	**0.001**
	No	74	226	1	1	1
Alcohol drinking	Yes	131	161	6.24 (3.86–10.12)	**4.59** **(2.60–8.12)**	**0.000**
	No	24	184	1	1	1
Khat chewing	Yes	39	43	2.36 (1.46–3.83)	1.53 (0.82–2.84)	0.182
	No	116	302	1	1	1
Watching Pornography	Yes	93	93	4.07 (2.73–6.06)	**2.59** **(1.59–4.23)**	**0.000**
	No	62	252	1	1	1
Night club	Yes	131	206	3.68 (2.28–5.99)	**2.75** **(1.47–5.16)**	**0.002**
	No	24	139	1	1	1
Social media	Yes	150	320	2.34 (0.89–6.24)	0.72 (0.21–2.48)	0.599
	No	5	25	1	1	1

* Indicated significant at *p* value <0.05 and values in bold represent statistically significant variables.

## Discussion

The results of the current study revealed that the prevalence of casual sex was 31.0% (27.2–34.6). These findings align with a study conducted among college students in Desse town, which reported a prevalence of 28% (7). However, this is higher than the 11.9% reported in a study conducted at Alkan University in Addis Ababa (9).

The discrepancy in these findings might be due to differences in how this study operationalized casual sex and the time period of the study. However, the prevalence is lower than that found in studies conducted in Taiwan (36.1%), (10) in Ohio (40%) (11), and in Los Angeles (54.8%) (12). The possible explanation for this difference could be the social, cultural, and religious differences in casual sexual activity between these three countries and Ethiopia. In developing countries, including Ethiopia, sexual activity without a romantic relationship is often viewed as shameful or unacceptable by society. Conversely, developed countries often view casual sex as a healthy sexual outlet, akin to regular exercise or an enjoyable physical experience (13).

Among the associated factors, the academic performance of the participants was significantly linked to casual sex. Students with a cumulative GPA of 2.5 or less were 2.81 times more likely to engage in casual sex than students with a cumulative GPA greater than 2.5. This finding is corroborated by a study conducted at the University of Southern California in the U.S.A. (14). This might be because students with good grades spend a significant amount of time on their studies. Conversely, students with poor academic performance may not attend classes regularly and may spend their time enjoying themselves rather than studying. Additionally, they might engage in casual sex to compensate for their negative academic performance (15, 16).

The results of this study revealed a significant association between alcohol consumption and casual sex. The odds of engaging in casual sex are four times greater for respondents who consume alcohol than for those who do not. This finding is corroborated by studies conducted in Los Angeles (12, 17). A possible explanation could be that consuming alcohol leads to intoxication, which in turn increases sexual arousal among university students. Consequently, they may make decisions without fully considering their sexual partners or recognizing the potential consequences of engaging in casual sex (18).

This study also showed that place of residence was another factor associated with casual sex. Students from urban areas were almost three times more likely to engage in casual sex than were those from rural areas. This might be because students from urban areas have access to mobile communication, frequent night clubs, and, which could easily stimulate their sexual desire and lead them to engage in casual sex. Conversely, students from rural areas might be less likely to be exposed to these influences (19–22).

Respondents who had ever experienced peer pressure to have sex were 2.2 times more at risk of engaging in casual sex than were their counterparts. This might be because young people are more likely to share their day-to-day experiences with their friends. This is particularly noticeable among university students who live away from their parents and thus have less parental supervision. Additionally, young people often seek attention and recognition from their peers, which may lead them to behave in ways that mirror the practices of their close friends (23, 24).

Respondents who had watched pornographic films were at a greater risk of engaging in casual sex. This may be due to access to advanced mobile technology, the internet, and the widespread portrayal of pornographic videos across the globe, which fuels the issue of risky sexual behavior, including casual sex, among young people. Furthermore, young people are prone to experimentation due to their natural transition to adulthood. As a result, they may be influenced by the pornographic videos they watch to experiment with someone whom they do not consider a romantic partner (25, 26).

This study indicated that attending. Students who frequent nightclubs might be more likely to consume alcohol, use substances, and engage in sex with casual partners (27, 28).

### Strength and limitation of the study

This study aims to reveal the determinants of casual sex in higher education settings, providing valuable insights for targeted interventions. One of the study's strengths lies in its adequate sample size, which enhances the generalizability of the findings. Due to the structure of the university program, no first-year students were included from the randomly selected departments. Consequently, data were collected from students in their second to fifth years. However, the study did not incorporate qualitative data collection methods to cross verify the findings.

## Conclusion

The prevalence of casual sex among Debre Berhan University students was high, at 31%. Casual sex was found to be significantly associated with factors such as cumulative GPA, residence, alcohol consumption, nightclub attendance, pornography viewing, and peer pressure. Measures to prevent this include improving students' academic performance, providing free and appealing entertainment facilities, blocking access to pornographic websites on the university's Wi-Fi, fostering an environment conducive to peer education, and recruiting peer educators. These are important preventative measures.

### Recommendations

Tutorial classes should be prepared for those with poor academic performance, and serious lecture attendance should be enforced. Universities should provide free, attractive entertainment facilities or services to prevent students from frequenting places that may expose them to casual sex. The “Gondar Family Project” should be adopted because it creates an opportunity for students to spend time with their second foster family. The university should restrict access to pornographic websites on the Wi-Fi campus to limit easy access for students. Finally, universities should foster an environment conducive to peer education and recruit peer educators. A link should be created between the youth-friendly service offices located at various health facilities in Debre Berhan town and the university clinic. These services should also be provided within the university compound. Research on casual sex, especially in our country, Ethiopia, has been limited. Therefore, it would be beneficial to conduct more studies. A qualitative study should be undertaken to explore why students engage in casual sex.

## Data Availability

The raw data supporting the conclusions of this article will be made available by the authors, without undue reservation.
